# The relationship between serum p53 autoantibodies and characteristics of human breast cancer.

**DOI:** 10.1038/bjc.1994.219

**Published:** 1994-06

**Authors:** B. Mudenda, J. A. Green, B. Green, J. R. Jenkins, L. Robertson, M. Tarunina, S. J. Leinster

**Affiliations:** Department of Surgery, Royal Liverpool University Hospital, UK.

## Abstract

**Images:**


Br. J. Cancer (1994), 69, 1115–1119 © Macmillan Press Ltd., 1994

### The relationship between serum p53 autoantibodies and characteristics of human breast cancer

B. Mudenda[1], J.A. Green[2], B. Green[3], J.R. Jenkins[4], L. Robertson[5], M. Tarunina[4] & S.J. Leinster[1]

*Departments of [1]Surgery, [2]Medicine and [3]Pathology, Royal Liverpool University Hospital, Liverpool L7 8XP, UK; [4]Marie Curie Cell Proliferation Laboratory, The Chart, Oxted, Surrey, L7 8XP, UK; [5]Clatterbridge Cancer Research Trust Laboratories, Clatterbridge Hospital, Bebington, Merseyside L63 4JY, UK.*

**Summary** Sera from 182 newly diagnosed breast cancer patients were assayed for antibodies to p53 using an enzyme-linked immunosorbent assay (ELISA) method, and antibodies were detected in 48 (26%) compared with 1 out of 76 (1.3%) normal control volunteers ($P = 0.0001$). In breast cancer patients, autoantibodies were found in all stages of disease progression: carcinoma *in situ*, primary invasive breast cancer and in metastatic disease. In the subset of patients in whom sequential sera were assessed over a 6 month period, changes in the p53 antibody titres were observed. The presence of antibodies to p53 correlated positively with high histological grade ($P = 0.0012$) and a history of second primary cancer (six positive out of eight cases). The incidence of autoantibodies was lower in those patients with a first-degree relative with breast cancer ($P = 0.046$). Out of 68 patients, there was a significant correlation between positive p53 autoantibody status and the detection of p53 protein in the tissue sections by immunocytochemistry ($P = 0.002$). In the seronegative patients, positive p53 tumour staining was strongly associated with a family history of breast cancer ($P = 0.009$). The p53 protein overexpressed in heritable breast cancers may therefore be less immunogenic. The presence of p53 autoantibodies provides important additional information to immunochemistry and may identify patients with aggressive histological types of breast cancer.

### Introduction

Inactivation of the p53 gene is the commonest genetic change in human malignancy, and may occur by mutation, complex formation with other cellular proteins or enhanced degradation (Nigro *et al.*, 1989; Hollstein *et al.*, 1991; Crook *et al.*, 1992; Oliner *et al.*, 1992). Mutation appears to be the commonest of these, occurring in between 30 and 50% of breast cancer patients (Davidoff *et al.*, 1991; Coles *et al.*, 1992), and gives rise to mutant p53 proteins of increased stability, which can be detected by immunohistochemistry. Germ-line mutations have been described in patients with the rare Li–Fraumeni syndrome, which includes breast cancer (Malkin *et al.*, 1990), but recent evidence would suggest germ-line mutations in p53 do not account for the majority of heritable breast cancers (Prosser *et al.*, 1991; Sidransky *et al.*, 1992). However, mechanisms of stabilisation other than mutation may give rise to immunohistochemically detectable protein in breast cancer families (Barnes *et al.*, 1992). Data from large clinical series of both early and metastatic breast cancer have shown a correlation between p53 protein expression and clinical outcome (Thor *et al.*, 1992; Allred *et al.*, 1993).

The occurrence of high levels of cellular mutant p53 often follows critical events in malignancies. For instance, haematopoietic cells of leukaemic patients in remission or in chronic phase have immunohistochemically undetectable p53 levels but exhibit high p53 levels upon relapse or when tumour cells enter blast crisis (Nakai *et al.*, 1992). Similarly, in cervical cancer, while p53 mutation may not be present in the majority of tumours in early disease, mutations have been described in lymph node metastases of human papillomavirus (HPV)-positive tumours. In breast cancer high nuclear levels of p53 are often associated with advanced disease, and correlate with known indicators of poor prognosis, such as tumour differentiation and low progesterone receptor concentrations (Cattoretti *et al.*, 1988). There is also variation in the distribution of p53 protein expression at different phases of the cell cycle, with accumulation at the $G_1$–S interface (Shaulsky *et al.*, 1990).

Autoantibodies to p53 have been detected in the serum of breast cancer patients (Crawford *et al.*, 1982; Davidoff *et al.*, 1992; Schlichtholz *et al.*, 1992) as well as in the serum of patients with lung and other tumour types (Caron de Fromentel *et al.*, 1987; Winter *et al.*, 1992). The current study was initiated to assess the clinical implications of serum antibodies to p53 by relating their incidence as determined by a novel ELISA technique to both tissue p53 protein expression in the corresponding biopsy and established clinicopathological variables in this disease.

Correspondence: J.A. Green.
Received 2 September 1993; and in revised form 26 January 1994.

### Patients and controls

All patients presenting for treatment of newly diagnosed breast cancer to the Breast Clinic at the Royal Liverpool University Hospital, Merseyside, UK, between December 1990 and August 1991 were recruited into this study after giving informed consent. Controls were derived from patients attending the Breast Clinic, with or without breast symptoms, known not to have suffered from any malignant disease and found not to have any active breast lesions following clinical and/or mammographic examination. These control patients were divided into two groups, those with and without a family history of breast cancer. Volunteers who had more than one first-degree relative with a malignant disease were also excluded. Patients and controls were interviewed and their laboratory and hospital records consulted and updated accordingly.

The age of the patients, parity (with multiple births treated as single full childbirth) and menopausal status were recorded. Premenopausal women were those still menstruating or, if they were hysterectomised without oophorectomy, aged 45 years or less. Post-menopausal status was entered when women had ceased menstruating regularly from natural causes or had undergone bilateral oophorectomy or ovarian irradiation without subsequent menstruation, or if they were hysterectomised without bilateral oophorectomy aged 55 or over. Menopausal status was recorded as unknown if the patient had a hysterectomy without oophorectomy and was aged between 45 and 55 years of age.

A history of malignant disease excluding skin neoplasia suffered by the patient was recorded, and a detailed family history of breast cancer and any other cancers of first-degree relatives (siblings, offspring, parents, aunts, uncles, grandparents) and second-degree relatives such as cousins was also recorded. Menarche, age of first childbirth and history of lactation were not considered.

Mammographic records when tumours were impalpable were recorded as mass; microcalcification or stromal (parenchymal) deformity; or a combination of these. Pathological results were recorded according to the original report given for clinical management, except for those in which tumour differentiation was assessed, which were subjected to a further review by the study pathologist.

### *Serum*

Blood was obtained and centrifuged within 2 h of collection. The serum was divided into aliquots of 1.5 ml and stored in cryovials at −20°C until required. Only one sample was taken from the control group. From the breast cancer group sera were collected as follows:

1. Patients with carcinoma *in situ* (CIS) and early breast cancer: at time of diagnosis, 6 weeks after primary treatment and 6 months after primary treatment.
2. Patients with advanced breast cancer: at time of diagnosis, 6–8 weeks after commencing chemotherapy and 6 months after last chemotherapy.

The breast excision specimens were fixed in 10% neutral buffered formalin for at least 18 h, inspected, measured, processed in paraffin wax and 5 μm sections stained with haematoxylin and eosin.

### Methods

The definitive study of the sera from 285 patients and controls was carried out using an ELISA technique, in which soluble p53 protein with deletion of 132 amino acids from the N-terminus was produced in the Marie Curie Institute by the polymerase chain reaction and cloning in the pDS/RSB bacterial expression plasmid (Gentz *et al.*, 1989). Identity of the polymerase chain reaction product to wild-type human p53 was confirmed by sequencing. The fragment of p53 was expressed in *Escherichia coli* and, after harvesting by centrifugation and lysis, the protein supernatant was purified on an Ni-NTA agarose column (Quiagen). Renaturation was by serial dialysis in 25 mM Tris–HCl buffer, pH 8, with decreasing concentrations of guanidine hydrochloride. After incubation, unbound p53 was removed by washing and 50 μl from each serum sample at a standard dilution of 1:200 was then added to each well in triplicate, after a blocking step with bovine serum albumin. Standard controls comprised a set of wells with the monoclonal antibody to p53 PAb 122 (Boehringer Mannheim) and a set of pooled positive sera and pooled negative sera from an initial series of samples assayed by immunoblotting. Peroxidase-conjugated rabbit anti-human secondary antibody to IgG was then added to each well, and the optical density was determined at 490 nm after reaction with OPD (Dako). As additional proof of the specificity of the method for antibodies to p53, preincubation of the positive control sera with the purified p53 protein caused a downward shift in the curve of optical density plotted against serum dilution in the range 1:100 to 1:1,000 for the controls positive by immunoblotting. Twelve sera (six ELISA positive, six ELISA negative) were tested for anti-nuclear antibody by indirect immunofluorescence on rat liver sections and HEP/2 cells, and against double-stranded DNA by ELISA. These assays were carried out in the Regional Immunology Laboratory, Royal Liverpool University Hospital, and subject to national quality control criteria. All 12 sera were negative.

In order to identify positive sera with p53 antibodies, sera from a separate series of 26 patients with breast cancer were assayed for these antibodies by a Western blotting method, similar in outline to that employed by Winter *et al.* (1992), and four (15.4%) were found to be positive. Full details of the technique are available from the authors. Monkey Cos-1 cells were transfected with the p53 gene (mutant and wild type) and the p53 protein immunoprecipitated at 48 h. This was purified on an SDS-PAGE gel, which was subsequently transferred to nitrocellulose filters and exposed to the patient's serum diluted at 1:50. Detection was based on further incubation of the filters with $^{125}$I-labelled protein A (Figure 1). Tissue sections from paraffin-embedded material on the corresponding biopsy were analysed by the avidin–biotin–immunoperoxidase method using CM1 polyclonal antibody to p53 protein supplied by D. Lane, University of Dundee, Scotland, UK, and a biotinylated swine anti-rabbit secondary antibody (Dako), according to the method of Hsu *et al.* (1988). Positive nuclear staining was recorded by comparison with Cos-1 cells formalin fixed and embedded in paraffin. Sections were classified as negative, weakly positive, positive (equal intensity to the positive control) or strongly positive. All categories other than negative were taken as positive for analysis.

The ELISA method was then validated by comparing the optical density plot of this series with that of the negative control, and the cut-off point was defined as 2.5 times the negative control. Two of the 22 patients negative by the Western technique were found to be positive by the ELISA method. The dilution titre of the six positive patients gave a linear curve within the dilution range 1:10 up to 1:5,000, and for the analysis of the test sera, serum diluted at 1:200 was used, a 4-fold greater dilution than employed in the immunoblotting method. Maximum detection of the p53 antibodies by the PAb 122 monoclonal was obtained at a p53 concentration of 3 μg ml$^{-1}$, 50 μl of which was applied to each well.

### *Statistical methods*

The precision of the ELISA method was tested using linear regression analysis. The relationship between individual and grouped clinical and pathological variables was tested by means of chi-square tests for contingency tables with ordered categories (Armitage & Berry, 1987). Multiple analyses were performed by means of multiple linear regression analysis. All the $P$-values used were two-sided, and $P$-values of less than 5% were judged to be statistically significant.

### Results

Serum antibodies to p53 were detected in 26% (48/182) of the sera of breast cancer patients, compared with 1/76 of the control patients ($P = 0.0001$). Of the 99 tumours graded according to the modified classification of Bloom and Field, (1971) (Table I), a significant positive correlation was found

**Figure 1** Western immunoblot analysis of a test series of breast and lung cancer sera. A 50% mixture of mutant (conserved box 5) and wild-type p53 is run on the gel and, after incubation with test serum and development with $^{131}$I-labelled protein A, a positive result is shown by a band at a molecular weight of 53 kDa. The positive control (lane 1) employs a mouse monoclonal antibody to human p53 instead of test serum.

between seropositivity and poor tumour differentiation ($P<0.0012$).

At least two serum samples were obtained at least 6 weeks apart in 22 of the 182 patients. In eight patients three or more serum samples were assayed over a period of 6 months or more. p53 antibody status did not change with time in serial serum samples. These findings include the results in the four patients who were found to have host antibodies to p53 by the immunoblotting method on the first sample, and who continued to be seropositive for antibodies by ELISA in their subsequent serial sera. Even in patients with *in situ* disease in whom the tumour had been surgically removed at least 6 months prior to the second sample, there was no significant change in serum titre. However, two patients who had been found negative by immunoblotting in the original series of 26 patients were found to be seropositive by ELISA.

Patients with high titres and high optical density absorbency at the beginning continued to exhibit high levels in subsequent sera. Similarly, those with lower or moderate titre levels maintained similar levels over 6 months. The relationship between p53 autoantibody status and the clinicopathological parameters of breast cancer is shown in Table II. In 40 patients the tumours were smaller than 2 cm, and in 114 patients they were 2 cm or more. In the remainder (28 patients) no tumour measurements were available. No obvious associations could be made between p53 host antibody status and either size of tumour ($P = 0.42$) or axillary lymph node metastases ($P = 0.435$). Sixty-two patients were free of axillary node metastases and 57 had lymph node metastases. All the tumours were characterised and typed, and 127 patients were classified as having ductal carcinoma of no specific type, 16 as having lobular carcinoma and 23 as having ductal carcinoma *in situ* (DCIS). There was no association between serological status and histological type. While eight of the DCIS patients were positive, there was no effect of the presence or absence of associated extensive DCIS in the biopsies.

Some of the subsets of metastatic and locally advanced breast cancer patients (Table II) showed a high prevalence of seropositivity (3/5 with bone metastases, 1/3 with lung and 4/9 locally advanced disease), but none of the four patients with liver metastases, of whom two were jaundiced, were found to have autoantibodies to p53, and of a total of 27 advanced breast cancer patients eight (29.6%) were found to be seropositive. There was no significant association with tumour stage. Table III demonstrates that a high Nottingham prognostic index was associated with the presence of serum p53 autoantibodies ($P = 0.03$). This index provides a score summating histological grade and extent of tumour spread (Ellis *et al.*, 1992).

The association of p53 autoantibody with the characteristics of the breast cancer patients is shown in Table IV. In a subset of women not subject to mammographic screening programmes (i.e. less than 50 years of age), 7 of 15 patients (47%) aged below 40 were found to be seropositive for autoantibodies to p53 compared with 18% (8/43) of patients aged between 40 and 49. Thirty per cent (14/46) of seropositive women were premenopausal and the majority (41/46)

**Table I** Correlation between p53 host antibody and histological grade of breast cancer

| *Grade* | *Negative* | *Positive* | *Total* |
|---|---|---|---|
| I | 21 | 1 (6%) | 22 |
| II | 42 | 7 (14%) | 49 |
| III | 36 | 22 (38%) | 58 |
| Not available | 20 | 10 (33%) | 30 |

$\chi^2 = 13.52$, $P<0.0012$. $\chi^2 = 12.77$, $P<0.0004$.

**Table II** The relationship between p53 autoantibody and clinicopathological parameters of breast cancer

| *Parameter* | *Negative* | *Positive* | *Total* |
|---|---|---|---|
| Size of tumour (cm) | | | |
| <2.0 | 32 | 8 (20%) | 40 |
| >1.9 | 82 | 32 (28%) | 114 |
| $\chi^2 = 0.63$, $P = 0.42$ | | | |
| Lymph node metastases | | | |
| None | 48 | 14 (22%) | 62 |
| 1-3 | 20 | 6 (23%) | 26 |
| >3 | 20 | 11 (35%) | 31 |
| Unknown | 31 | 9 (22%) | 40 |
| $\chi^2 = 2.18$, $P = 0.53$; $\chi^2$ for trend = 1.57, $P = 0.21$ (0→3 metastases) | | | |
| Distant metastases | | | |
| Lung and pleural | 3 | 1 | 4 |
| Liver | 4 | 0 | 4 |
| Bone | 2 | 3 | 5 |
| Meningeal | 1 | 0 | 1 |
| Locally advanced | 9 | 4 | 13 |
| Total | 19 | 8 | 27 |
| Stage of breast cancer | | | |
| Stage I | 17 | 2 (10%) | 19 |
| Stage II | 60 | 19 (24%) | 79 |
| Stage III | 19 | 5 (20%) | 24 |
| Stage IV | 12 | 7 (36%) | 19 |
| Not available | 8 | 5 (31%) | 13 |
| $\chi^2 = 3.778$, $P = 0.2864$. | | | |
| $\chi^2$ for trend = 2.535, $P = 0.114$. | | | |

**Table III** Nottingham prognostic index (Ellis *et al.*, 1992)

| | *Negative* | *Positive* | *Total* |
|---|---|---|---|
| <3.4 | 41 | 7 (14%) | 48 |
| 3.4-5.4 | 22 | 11 (33%) | 33 |
| >5.4 | 31 | 16 (34%) | 47 |
| Unknown | 20 | 6 (23%) | 26 |

$\chi^2 = 5.655$, $P = 0.059$; $\chi^2$ for trend = 4.633, $P = 0.0314$.

**Table IV** The association of p53 autoantibody and the personal clinical history of breast cancer patients

| | *Negative* | *Positive* | *Total* |
|---|---|---|---|
| Age at diagnosis (years) | | | |
| <30 | 1 | | 1 |
| 30-39 | 7 | 7 (50%) | 14 |
| 40-49 | 34 | 8 (18%) | 43 |
| 50-59 | 38 | 17 (30%) | 55 |
| 60-69 | 32 | 9 (21%) | 43 |
| 70-79 | 13 | 6 (31%) | 19 |
| >79 | 4 | 1 (20%) | 5 |
| $\chi^2 = 6.34$, $P = 0.274$; $\chi^2$ for trend = 0.406, $P = 0.52$ | | | |
| Parity | | | |
| Nulliparous | 26 | 12 (30%) | 38 |
| Parous | 75 | 30 (27%) | 105 |
| Unknown | 32 | 7 (11%) | 39 |
| $\chi^2 = 0.122$, $P = 0.7272$ | | | |
| Menstrual status | | | |
| Premenopausal | 32 | 14 (30%) | 46 |
| Post-menopausal | 87 | 32 (27%) | 121 |
| Unknown | 17 | 2 (10%) | |
| $\chi^2 = 0.207$, $P = 0.6489$ | | | |
| Previous malignancy | | | |
| other than breast | 2 | 6 (75%) | 8 |
| Number of first-degree relatives with cancer | | | |
| Breast cancer | 30 | 3 (11%) | 33 |
| Two or more cancers | 16 | 6 (37%) | 22 |
| $\chi^2 = 2.00$, $P = 0.157$ (Fisher's exact $P = 0.079$) | | | |

**Figure 2** Breast cancer section stained with the polyclonal CMI antibody to human p53 and developed by the avidin–biotin–immunoperoxidase method. Positive nuclear staining is observed (× 200).

**Table V** Correlation between p53 host antibody and p53 staining in breast tumours

| | *Negative* | *Positive* |
|---|---|---|
| Seropositive ($n$ = 23) | 5 | 18 (78%) |
| Seronegative ($n$ = 45) | 29 | 16 (35.5%) |

$\chi^2 = 9.46, P < 0.002$.

were symptomatic. Of the premonopausal seropositive women, 42% (6/14) seropositive patients were found to have grade 3 tumours.

Figure 2 shows an example of positive nuclear staining for p53 in a breast carcinoma. When the presence of autoantibodies in the sera by the ELISA technique was compared with immunocytochemical localisation of p53 protein in 68 corresponding tumours, there was good correlation ($P = 0.002$) between seropositivity and the presence of p53 in tumours (Table V). In the seronegative patients the overexpression of p53 in the tumour biopsies was found to correlate positively with a family history ($P = 0.009$). Six (75%) out of eight patients who had suffered from another primary cancer apart from breast cancer (patients with a second primary breast tumour were not included) were found to exhibit p53 host antibodies in their serum. Three patients had both the primary tumours immunohistochemically stained for p53, and in two of these evidence of p53 overexpression was found in both.

A family history of two or more relatives with cancers other than breast cancer also appeared to be significantly associated with seropositivity for p53 autoantibodies. However, only 3 out of 33 (9%) breast cancer patients with a family history of breast cancer were found to have autoantibodies to p53, compared with 42 out of 143 breast cancer patients without a family history who were seropositive ($P = 0.046$). All the three seropositive were patients with only one relative with breast cancer, and none of the seven breast cancer patients with two or more relatives with breast cancer exhibited autoantibodies to p53.

### Discussion

This study has demonstrated a higher prevalence (26%) of serum autoantibodies to p53 protein than previously demonstrated by immunoblotting techniques which have shown prevalence rates between 11 and 15% (Crawford *et al.*, 1982, 1984; Caron de Fromentel *et al.*, 1987; Davidoff *et al.*, 1992; Winter *et al.*, 1992). This high prevalence approaches that of the value for p53 mutation (*c.* 30%) in breast cancer. However, it is likely that mutation is only one mechanism of inactivation of p53. The most significant observation was the association with poor histological grade, as has also been noted in one recent study (Schlichtholz *et al.*, 1992). There was also a good correlation between seropositive status and a high Nottingham prognostic index. The presence of p53 autoantibodies has also been shown to be associated with the subset of p53 mutants which bind tightly to heatshock protein 70 (Davidoff *et al.*, 1992). Such mutations have strong transforming properties *in vitro*, and this would be consistent with their presence in tumours of poor histological grade.

The relationship to metastatic disease in the present study was not clear, with a trend in favour of a higher prevalence in more advanced disease, as was noted by Crawford (1984) in his original series. However, Schlichtholz *et al.* (1992) found no association with metastases, and therefore suggested that the appearance of antibodies was an early event. In gastric cancer p53 overexpression may be associated with a high potential for metastasising to the lymph nodes in other tumour types (Kakeji *et al.*, 1993), and in cervical cancer overexpression is common in HPV-negative tumours which have a poor prognosis (Crook *et al.*, 1992).

There is also conflicting evidence on whether the autoantibodies recognise mutant or wild-type p53. De Leo *et al.* (1979) showed in experimental systems that the mutant p53 may be immunogenic. However, Davidoff *et al.* (1992) showed in breast cancer that p53 autoantibodies recognise a variety of wild-type and mutant p53 molecules. Schlichtholz *et al.* (1992), on the other hand, using fusion proteins from parts of the p53 molecule, showed that the antibodies were recognising sections of the protein near the C- and N-termini of the molecules, and well away from the mutational hotspot region in the evolutionarily conserved boxes in exons 4–8 (Winter *et al.*, 1992) in patients with non-small-cell lung cancer, and Lebrecque *et al.* (1993) in colon and breast cancer demonstrated that the antibodies recognised both wild-type and mutant p53 conformational and denaturation-resistant epitopes.

The p53 protein employed in the present ELISA method (but not that in the immunoblotting techniques) is lacking part of the N-terminal peptide, and therefore these studies may under-represent the true incidence of serum autoantibodies in breast cancer patients.

The lower prevalence of autoantibodies in the patients with a family history of breast cancer in the present study is of interest as it may represent loss of tolerance induced by strong accumulation of wild-type protein in the tumour cell, a hypothesis advanced by Schlichtholz *et al.* (1992). Against this hypothesis is the demonstration of relatively high levels of protein in the corresponding biopsies of seropositive patients, which supports the converse hypothesis, that mutant p53, provisionally of germ-line origin, gives rise to the production of antibodies specific to a protein determinant altered by mutation.

Thor *et al.* (1992) made the observation that 42% of 76 hereditary breast cancers showed positive tissue staining for p53, with approximately similar proportions of positive staining in breast and breast/ovarian cancer families. In the present series the majority of patients who showed tissue p53 overexpression were also found to be seropositive. There were also a number of patients who were positive by ELISA yet negative by immunocytochemistry. This observation was also made by Crawford *et al.* (1984), who found that 8 of 14 breast cancer patients positive for autoantibodies had no detectable p53 in their biopsies. It is suggested that in these cases the immunogenic stimulus may have occurred at an earlier stage of tumour development, perhaps when a higher proportion of tumour cells were in cycle, corresponding to a period of intense proliferative activity.

These observations suggest that serological analysis provides an assessment of the functional state of the p53 gene in breast cancer patients, and may prove to be a useful adjunct to molecular and histochemical methods of tumour characterisation which have to date concentrated on allele loss, gene mutation and protein expression. The correlation with poor histological differentiation suggests an association with adverse biological features.

The authors would like to thank Dr R. Barnes, Consultant Immunologist, Royal Liverpool University Hospital, for assistance with the antinuclear antibody analyses, and Mrs J. Eccles for expert typing of the manuscript.

### References

ALLRED, D.C., CLARK, G.M., ELLEDGE, R. FUQUA, S.A.W., BROWN, R.W., CHAMNESS, G.C., OSBORNE KENT, C. & MCGUIRE, W.L. (1993). Association of p53 protein expression with tumour cell proliferation rate and clinical outcome in node-negative breast cancer. *J. Natl Cancer Inst.*, **85**, 200-206.

ARMITAGE, P., BERRY, G. (1987). *Statistical Methods in Medical Research*, 2nd revised edn. Blackwell Scientific Publications: Oxford.

BARNES, D.M., HANBY, A.M., GILLETT, C.E., MOHAMMED, S., HODGSON, S., BOBROW, L.G., LEIGH, I.M., PURKIS, T., MACGEOCH, C., SPURR, N.K., BARTEK, J., VOJTESEK, B., PICKSLEY, S.M. & LANE, D.P. (1992). Abnormal expression of wild type p53 protein in normal cells of a cancer family patient. *Lancet*, **340**, 259-263.

BLOOM, H.J.G. & FIELD, J.R. (1971). Impact of tumour grade and host resistance on survival of women with breast cancer. *Cancer*, **28**, 1580-1589.

CARON DE FROMENTEL, C., MAY-LEVEN, F., MOURIESSE, H., LEMERLE, J., CHANDRASEKARAN, K. & MAY, P. (1987). Presence of circulating antibodies against cellular protein p53 in a notable proportion of children with B-cell lymphoma. *Int. J. Cancer*, **39**, 185-189.

CATTORETTI, G., RILKE, F., ANDREOLA, S., D'AMATO, L.D. & DELIA, D. (1988). p53 expression in breast cancer. *Int. J. Cancer*, **41**, 178-183.

COLES, C., CONDIE, A., CHETTY, U., STEEL, C.M., EVANS, H.J. & PROSSER, J. (1992). p53 Mutations in breast cancer. *Cancer Res.*, **52**, 5291-5298.

CRAWFORD, L.V., PIM, D. & LAMB, P. (1984). The cellular protein p53 in human tumours. *Mol. Biol. Med.*, **2**, 261-272.

CRAWFORD, L.V., PIM, D.C. & BULBROOK, R.D. (1982). Detection of antibodies against the cellular protein p53 in sera from patients with breast cancer. *Int. J. Cancer*, **30**, 403-408.

CROOK, T., WREDE, D., TIDY, J.A., MASON, W.P., EVANS, D.J. & VOUSDEN, K.H. (1992). Clonal p53 mutations in primary cervical cancer: association with human papillomavirus negative tumours. *Lancet*, **339**, 1070-1073.

DAVIDOFF, A.M., IGLEHART, J.D. & MARKS, J.R. (1992). Immune response to p53 is dependent upon p53/HSP70 complexes in breast cancer. *Proc. Natl Acad. Sci. USA*, **89**, 3439-3442.

DE LEO, A.B., JAY, G., APPELLA, E., DUBOIS, G.C., LAW, L.W. & OLD, L.J. (1979). Detection of a transformation related antigen in chemically induced sarcomas and other transformed cells of the mouse. *Proc. Natl Acad. Sci. USA*, **76**, 2420-2424.

ELLIS, I.O., GALEA, M., BROUGHTON, N., LOCKYER, A., BLAMEY, R.W. & ELSTON, C.W. (1992). Pathological prognostic factors in breast cancer. II. Histological type. Relationship with survival. *Histopathology*, **20**, 479-489.

GENTZ, R., CHEN, C.H. & ROSEN, C.A. (1989). Bioassay for transactivation using purified human immunodeficiency virus rat-encoded protein: transactivation requires mRNA synthesis. *Proc. Natl Acad. Sci. USA*, **86**, 825-824.

HOLLSTEIN, M., SIDRANSKY, D., VOGELSTEIN, B. & HARRIS, C.C. (1991). p53 mutations in human cancers. *Science*, **25**, 49-53.

HSU, S.H., RAINE, L. & FRAZER, H. (1988). Use of avidin-biotin peroxidase complex (ABC) in immunoperoxidase techniques: a comparison between ABC and unlabelled antibody (PAP) procedures. *J. Histochem. Cytochem.*, **19**, 577-579.

KAKEJI, D., KORENAGA, S. & TSUJITANI, H. (1993). Gastric cancer with p53 overexpression has high potential for metastasising to lymph nodes. *Br. J. Cancer*, **67**, 589-593.

LEBRECQUE, S., NAOR, N., THOMSON, D. & MATLASHEWSKI, G. (1993). Analysis of the anti-p53 antibody response in cancer patients. *Cancer Res.*, **53**, 3468-3471.

MALKIN, D., LI, F.P. & STRONG, L.C. (1990). Germ line p53 mutations in a familial syndrome of breast cancer, sarcomas, and other neoplasms. *Science*, **250**, 1233-1238.

NAKAI, H., MISAWA, S., TOGUCHIDA, J., YANDELL, D.W. & ISHIZAKI, K. (1992). Frequent p53 gene mutations in blast crisis of chronic myelogenous leukemia, especially in myeloid crisis harboring loss of a chromosome 17p[1]. *Cancer Res.*, **52**, 6588-6593.

NIGRO, J.M., BAKER, S.J., PREISINGER, A.C., JESSUP, J.M., HOSTETTER, R., CLEARY, K., BIGNER, S.H., DAVIDSON, N., BAYLIN, S. & DEVILEE, P. (1989). Mutations in the p53 gene occur in diverse human types. *Nature*, **342**, 705-708.

OLINER, J.D., KINZLER, K.W., MELTZER, P.S., GEORGE, D.L. & VOGELSTEIN, B. (1992). Amplification of a gene encoding a p53-associated protein in human sarcomas. *Nature*, **358**, 80-83.

PROSSER, J., ELDER, P.A., CONDIE, A., MACFADYEN, I., STEEL, C.M. & EVANS, H.J. (1991). Mutations in p53 do not account for heritable breast cancer: a study in five affected families. *Br. J. Cancer*, **63**, 181-184.

SIDRANSKY, D., TOKINO, T. & HELZLSOUER, K. (1992). Inherited p53 gene mutations in breast cancer. *Cancer Res.*, **52**, 2984-2986.

SCHLICHTHOLZ, B., LEGROS, Y., GILLET, D., GAILLARD, C., MARTY, M., LANE, D., CALVO, F. & SOUSSI, T. (1992). The immune response to p53 in breast cancer patients is directed against immunodominant epitopes unrelated to the mutational hot spot. *Cancer Res.*, **52**, 6380-6384.

SHAULSKY, G., BEN-ZE'EV, A. & ROTTER, V. (1990). Subcellular distribution of the p53 protein during the cell cycle of Balb/c 3T3 cells. *Oncogene*, **5**, 1707-1711.

THOR, A.D., MOORE, II, D.H., EDGERTON, S.M., KAWASAKI, E.S., REIHSAUS, E., LYNCH, H.T., MARCUS, J.N., SCHWARTZ, L., CHEN, L.-C., MAYALL, B.H. & SMITH, H.S. (1992). Accumulation of p53 tumor suppressor gene protein: an independent marker of prognosis in breast cancers. *J. Natl Cancer Inst.*, **84**, 845-855.

WINTER, S.F., MINNA, J.D., JOHNSON, B.E., TAKAHASHI, T., GAZDAR, A.F. & CARBONE, D.P. (1992). Development of antibodies against p53 in lung cancer patients appears to be dependent on the type of p53 mutation. *Cancer Res.*, **52**, 4168-4174.


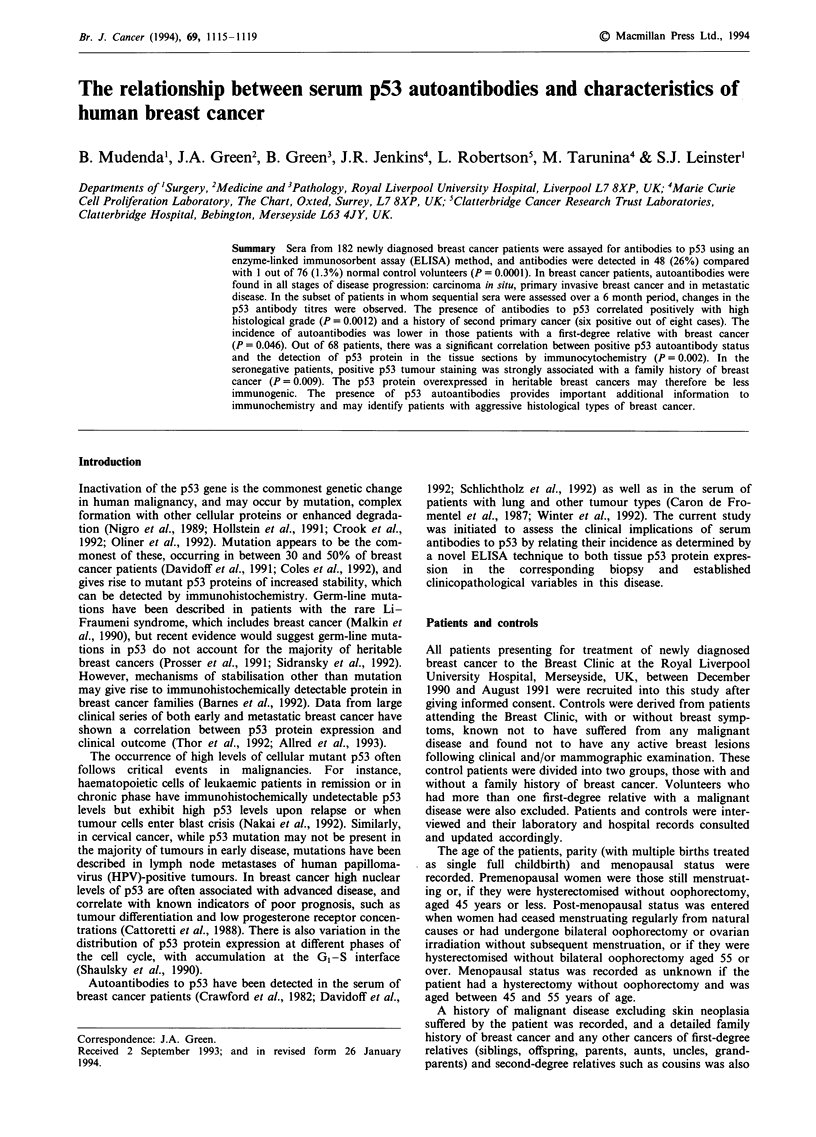

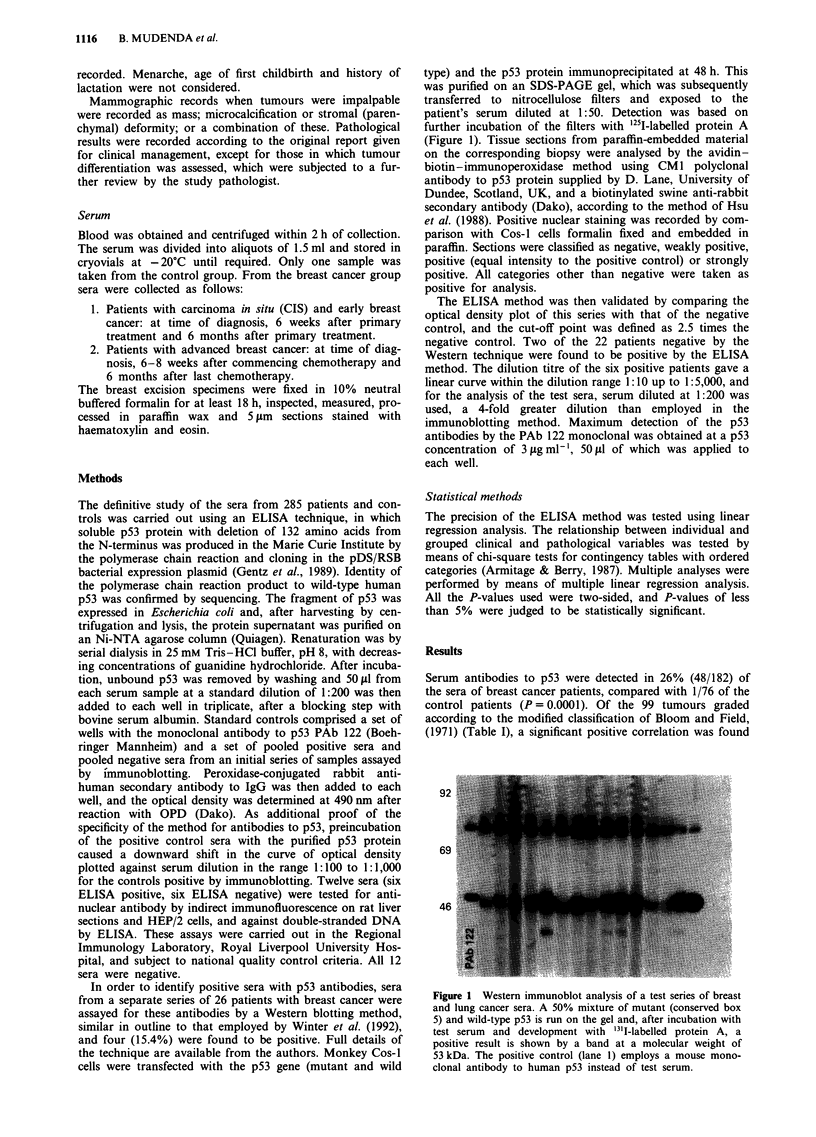

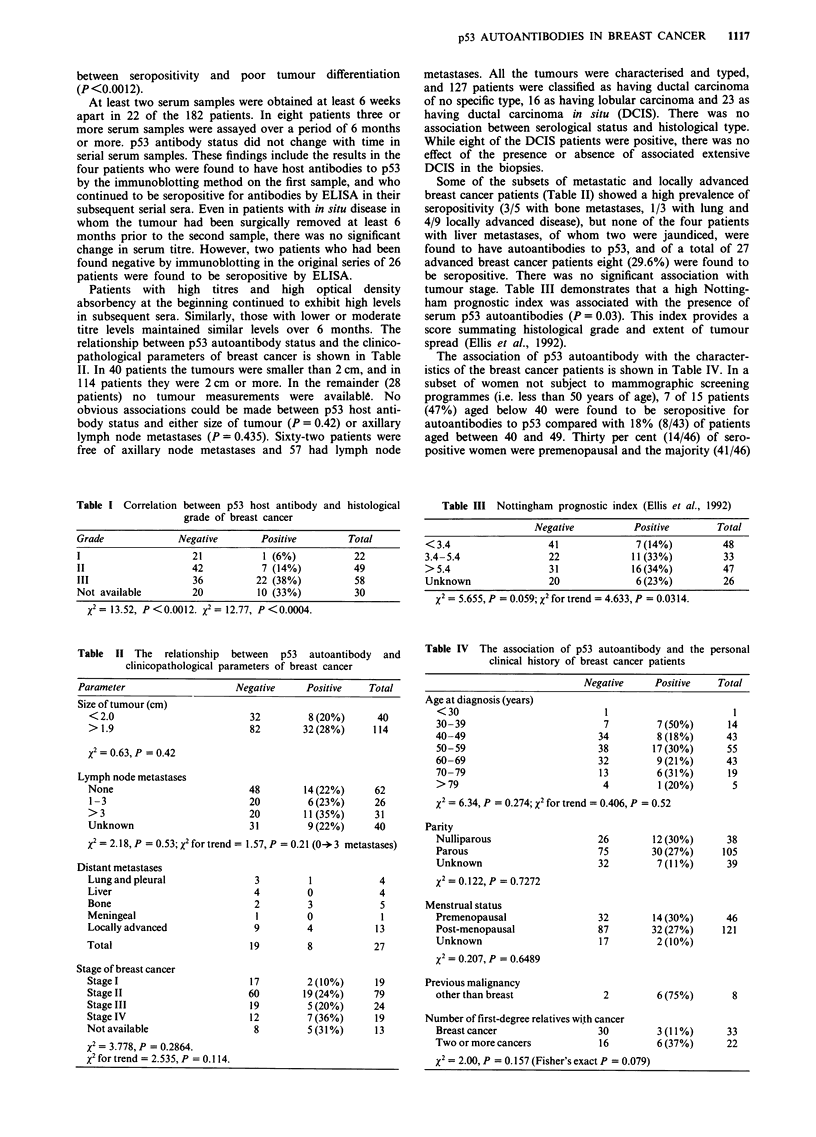

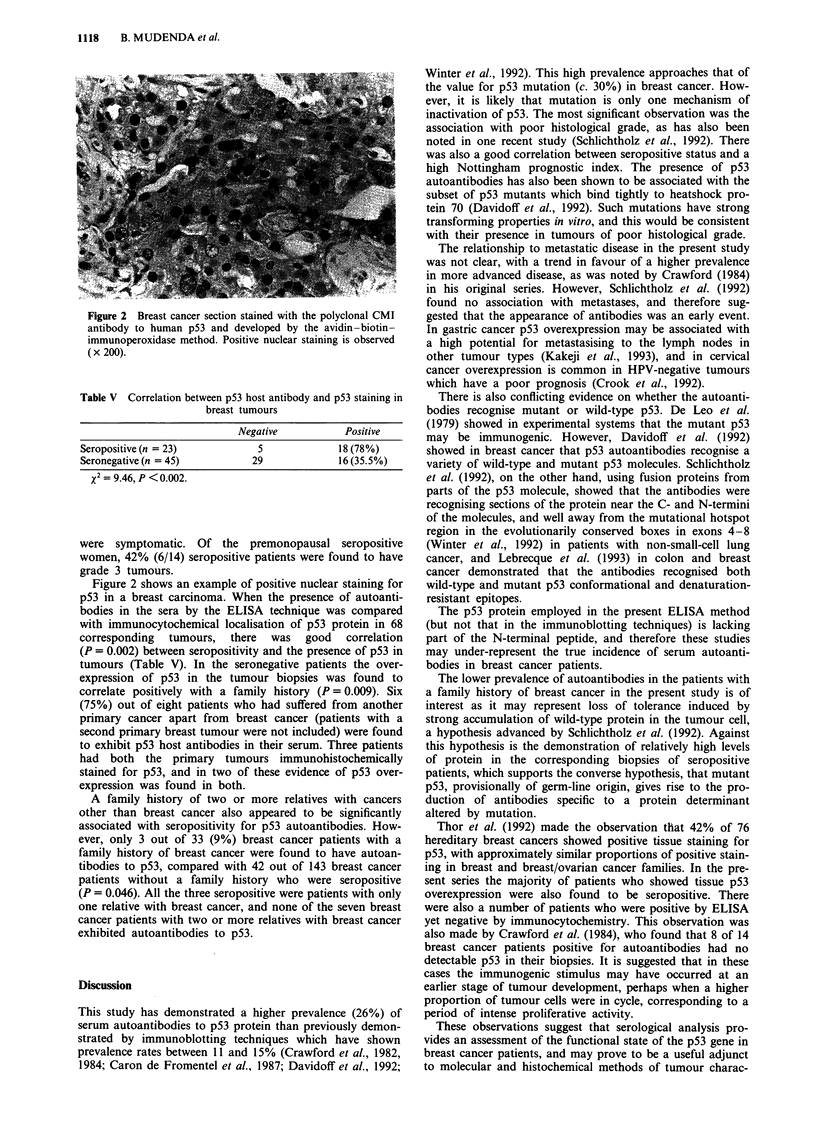

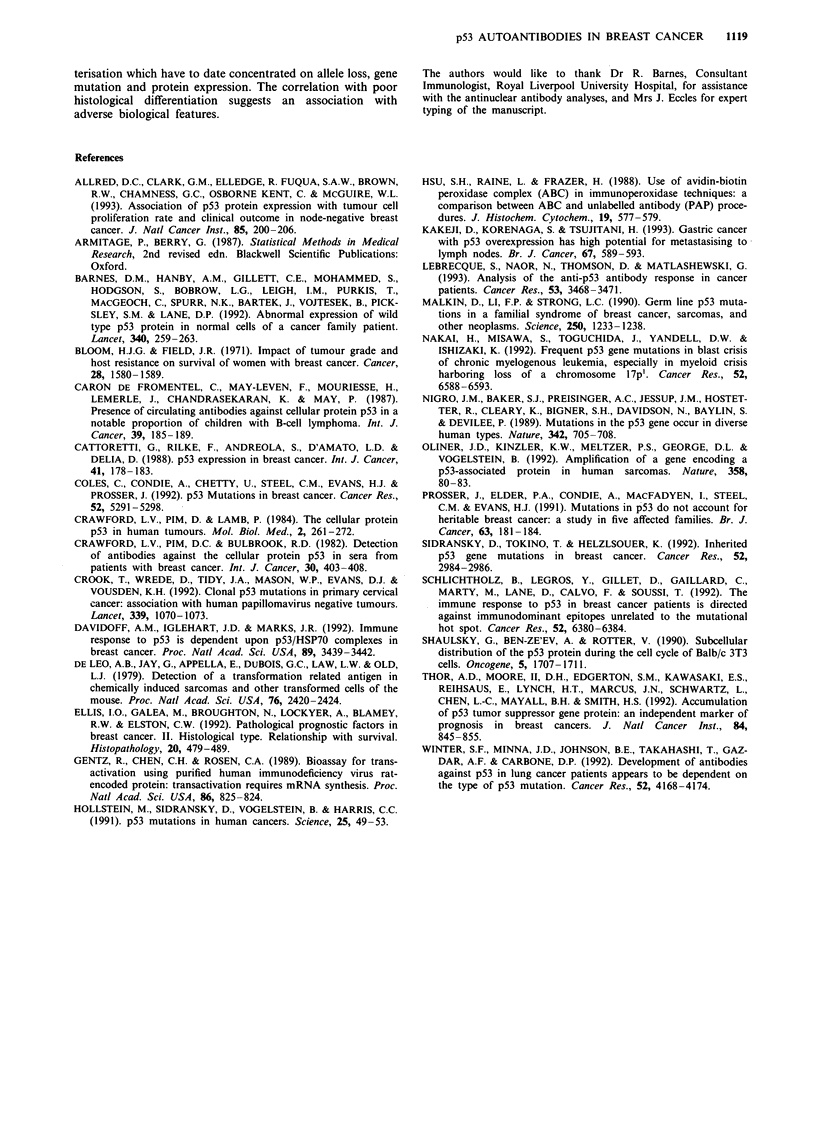


## References

[OCR_00660] Allred D. C., Clark G. M., Elledge R., Fuqua S. A., Brown R. W., Chamness G. C., Osborne C. K., McGuire W. L. (1993). Association of p53 protein expression with tumor cell proliferation rate and clinical outcome in node-negative breast cancer.. J Natl Cancer Inst.

[OCR_00674] Barnes D. M., Hanby A. M., Gillett C. E., Mohammed S., Hodgson S., Bobrow L. G., Leigh I. M., Purkis T., MacGeoch C., Spurr N. K. (1992). Abnormal expression of wild type p53 protein in normal cells of a cancer family patient.. Lancet.

[OCR_00678] Bloom H. J., Field J. R. (1971). Impact of tumor grade and host resistance on survival of women with breast cancer.. Cancer.

[OCR_00685] Caron de Fromentel C., May-Levin F., Mouriesse H., Lemerle J., Chandrasekaran K., May P. (1987). Presence of circulating antibodies against cellular protein p53 in a notable proportion of children with B-cell lymphoma.. Int J Cancer.

[OCR_00690] Cattoretti G., Rilke F., Andreola S., D'Amato L., Delia D. (1988). P53 expression in breast cancer.. Int J Cancer.

[OCR_00695] Coles C., Condie A., Chetty U., Steel C. M., Evans H. J., Prosser J. (1992). p53 mutations in breast cancer.. Cancer Res.

[OCR_00704] Crawford L. V., Pim D. C., Bulbrook R. D. (1982). Detection of antibodies against the cellular protein p53 in sera from patients with breast cancer.. Int J Cancer.

[OCR_00700] Crawford L. V., Pim D. C., Lamb P. (1984). The cellular protein p53 in human tumours.. Mol Biol Med.

[OCR_00709] Crook T., Wrede D., Tidy J. A., Mason W. P., Evans D. J., Vousden K. H. (1992). Clonal p53 mutation in primary cervical cancer: association with human-papillomavirus-negative tumours.. Lancet.

[OCR_00715] Davidoff A. M., Iglehart J. D., Marks J. R. (1992). Immune response to p53 is dependent upon p53/HSP70 complexes in breast cancers.. Proc Natl Acad Sci U S A.

[OCR_00720] DeLeo A. B., Jay G., Appella E., Dubois G. C., Law L. W., Old L. J. (1979). Detection of a transformation-related antigen in chemically induced sarcomas and other transformed cells of the mouse.. Proc Natl Acad Sci U S A.

[OCR_00726] Ellis I. O., Galea M., Broughton N., Locker A., Blamey R. W., Elston C. W. (1992). Pathological prognostic factors in breast cancer. II. Histological type. Relationship with survival in a large study with long-term follow-up.. Histopathology.

[OCR_00732] Gentz R., Chen C. H., Rosen C. A. (1989). Bioassay for trans-activation using purified human immunodeficiency virus tat-encoded protein: trans-activation requires mRNA synthesis.. Proc Natl Acad Sci U S A.

[OCR_00738] Hollstein M., Sidransky D., Vogelstein B., Harris C. C. (1991). p53 mutations in human cancers.. Science.

[OCR_00748] Kakeji Y., Korenaga D., Tsujitani S., Baba H., Anai H., Maehara Y., Sugimachi K. (1993). Gastric cancer with p53 overexpression has high potential for metastasising to lymph nodes.. Br J Cancer.

[OCR_00753] Labrecque S., Naor N., Thomson D., Matlashewski G. (1993). Analysis of the anti-p53 antibody response in cancer patients.. Cancer Res.

[OCR_00758] Malkin D., Li F. P., Strong L. C., Fraumeni J. F., Nelson C. E., Kim D. H., Kassel J., Gryka M. A., Bischoff F. Z., Tainsky M. A. (1990). Germ line p53 mutations in a familial syndrome of breast cancer, sarcomas, and other neoplasms.. Science.

[OCR_00763] Nakai H., Misawa S., Toguchida J., Yandell D. W., Ishizaki K. (1992). Frequent p53 gene mutations in blast crisis of chronic myelogenous leukemia, especially in myeloid crisis harboring loss of a chromosome 17p.. Cancer Res.

[OCR_00772] Nigro J. M., Baker S. J., Preisinger A. C., Jessup J. M., Hostetter R., Cleary K., Bigner S. H., Davidson N., Baylin S., Devilee P. (1989). Mutations in the p53 gene occur in diverse human tumour types.. Nature.

[OCR_00776] Oliner J. D., Kinzler K. W., Meltzer P. S., George D. L., Vogelstein B. (1992). Amplification of a gene encoding a p53-associated protein in human sarcomas.. Nature.

[OCR_00782] Prosser J., Elder P. A., Condie A., MacFadyen I., Steel C. M., Evans H. J. (1991). Mutations in p53 do not account for heritable breast cancer: a study in five affected families.. Br J Cancer.

[OCR_00793] Schlichtholz B., Legros Y., Gillet D., Gaillard C., Marty M., Lane D., Calvo F., Soussi T. (1992). The immune response to p53 in breast cancer patients is directed against immunodominant epitopes unrelated to the mutational hot spot.. Cancer Res.

[OCR_00800] Shaulsky G., Ben-Ze'ev A., Rotter V. (1990). Subcellular distribution of the p53 protein during the cell cycle of Balb/c 3T3 cells.. Oncogene.

[OCR_00788] Sidransky D., Tokino T., Helzlsouer K., Zehnbauer B., Rausch G., Shelton B., Prestigiacomo L., Vogelstein B., Davidson N. (1992). Inherited p53 gene mutations in breast cancer.. Cancer Res.

[OCR_00807] Thor A. D., Moore DH I. I., Edgerton S. M., Kawasaki E. S., Reihsaus E., Lynch H. T., Marcus J. N., Schwartz L., Chen L. C., Mayall B. H. (1992). Accumulation of p53 tumor suppressor gene protein: an independent marker of prognosis in breast cancers.. J Natl Cancer Inst.

[OCR_00815] Winter S. F., Minna J. D., Johnson B. E., Takahashi T., Gazdar A. F., Carbone D. P. (1992). Development of antibodies against p53 in lung cancer patients appears to be dependent on the type of p53 mutation.. Cancer Res.

